# Evidence From the Decade of Action for Road Safety: A Systematic Review of the Effectiveness of Interventions in Low and Middle-Income Countries

**DOI:** 10.3389/phrs.2022.1604499

**Published:** 2022-02-21

**Authors:** Maryam Tavakkoli, Zahra Torkashvand-Khah, Günther Fink, Amirhossein Takian, Nino Kuenzli, Don de Savigny, Daniel Cobos Muñoz

**Affiliations:** ^1^ Epidemiology and Public Health Department, Swiss Tropical and Public Health Institute (Swiss TPH), Basel, Switzerland; ^2^ University of Basel, Basel, Switzerland; ^3^ National Institute for Medical Research Development, Tehran, Iran; ^4^ School of Public Health, Tehran University of Medical Sciences, Tehran, Iran

**Keywords:** systematic review, accidents, traffic, wounds and injuries, systems analysis, low and middle income, road safety, decade of action

## Abstract

**Objectives:** To evaluate the effectiveness of road safety interventions in low and middle-income countries (LMICs), considering the principles of systems theory presented in the Global Plan for the Decade of Action for Road Safety.

**Methods:** We conducted a systematic review according to PRISMA guidelines. We searched for original research studies published during 2011–2019 in the following databases: Medline, Embase, PsycInfo, Scopus, Web of Science, Cochrane library, Global Health Library, ProQuest and TRID. We included studies conducted in LMICs, evaluating the effects of road traffic safety interventions and reporting health-related outcomes.

**Results:** Of 12,353 non-duplicate records, we included a total of 33 studies. Most interventions were related to legislation and enforcement (n = 18), leadership (n = 5) and speed management (n = 4). Overall, legislation and enforcement interventions appear to have the largest impact. Few studies were found for road infrastructure, vehicle safety standard and post crash response interventions.

**Conclusion:** Based on the currently available evidence, legislation and enforcement interventions appear most impactful in LMICs. However, many interventions remain understudied and more holistic approaches capturing the complexity of road transport systems seem desirable.

**Systematic Review Registration:** https://www.crd.york.ac.uk/prospero/display_record.php?RecordID=197267, identifier CRD42020197267.

## Introduction

With over 1.5 million deaths annually, road traffic injuries now account for the sixth highest cause of disability-adjusted life years lost globally (1). Road safety has become a major public health concern in many countries, resulting in increased attention from the international community. The United Nations integrated road safety into two of the 17 sustainable development goals (SDGs), SDG 3 and SDG 11 (2), and proclaimed 2011 to 2020 the Decade of Action for Road Safety (3).

Traditional approaches failed to capture the complexity of the road traffic systems. To address this, key principles of systems thinking were adopted in the UN Global Plan for the Decade of Action (3, 4). The plan provides an overall framework for countries to improve road safety (3) and is based on five pillars—road safety management, safer roads and mobility, safer vehicles, making road users safer, and improved post-crash response and hospital care—encouraging countries to pay more attention to vulnerable road users and shifting the responsibility of incidents from road users to the designers of the transport system (3).

Despite the increased global attention and progress in policy-making at national level, the number of road casualties increased in 87 low- and middle-income countries (LMIC) since 2013 (5, 6). LMICs bear more than 90% of deaths due to road traffic injuries, despite containing only 60% of the world’s motor vehicles. Death rates due to road traffic injuries in LMICs are three times higher than in high-income countries (HIC) (27.5 vs. 8.3 per 100,000 population) (6).

Even though findings about effective interventions from high-income countries (HIC) are potentially transferable to low resource settings, different context-specific factors such as costs, feasibility and barriers to implementation should be taken into account (7). Also, the traffic mix in LMICs is different than in HICs; the majority of evidence coming from HICs focuses on preventing injuries to vehicle occupants, but pedestrians and motorcyclists are the most vulnerable road users in LMIC settings (8).

During the past decade, few studies reviewed the effectiveness of road safety interventions in LMICs, however the results were limited to single interventions mostly in legislation and law enforcement category (9–11). A systematic understanding of interactions among different components of the system contributing to road safety is still lacking and very little is known about other road safety components, such as speed management, improving vehicle design, road infrastructure and post-crash response and their interactions in the system (12).

Following the end of the Decade of Action for Road Safety in 2020, we systematically reviewed the recent evidence on road safety interventions in LMICs. Our aim was to evaluate the effectiveness of road safety interventions in LMIC settings through the lens of the principles of the UN’s Global Plan for the Decade of Action.

## Methods

### Search Strategy and Selection Criteria

We conducted a systematic review and a narrative synthesis based on PRISMA guidelines as described in Supplementary Appendix A1 (13, 14). We included original research studies evaluating quantitative effects of road traffic safety interventions implemented in LMICs (5), reporting mortality, injury or crash as the primary outcome, online publication during January 2011–August 2019 in English, French, Spanish, Portuguese and Persian.

We used the World Bank income group classifications for fiscal year 2019, in which 138 LMICs are stratified by GNI per capita: low-income (under $1026), lower-middle income ($1026–$3,995) and upper middle-income ($3,996–$12,375) (5).

We developed a search strategy including terms to identify population, intervention, outcome and keywords using five pillars of the Global Plan as a framework (3). Then we used synonyms and variants of search terms to find relevant articles (15) in nine electronic databases including Medline, Embase, PsycInfo, Scopus, Web of Science, Cochrane library, Global Health Library, ProQuest and Transport Research International Documentation (TRID). We tested our search strategy by checking the inclusion of ten relevant studies in the field in our Medline search; then we modified the search for other databases as shown in Supplementary Appendix A2 Database search and retrieval of results from each database were done in August 2019.

After importing references to EndNote X9, we followed seven steps of deduplication in EndNote recommended by Bramer et al. (16). Then study authors (MT and ZT) independently conducted screening of titles, abstracts and full texts. We recorded the reasons for exclusion of studies and resolved disagreements by discussion to reach a consensus, or deferred to a third author (DC) for the remaining cases. We registered the protocol for our systematic review in the PROSPERO database (CRD42020197267).

### Data Synthesis

The primary outcome measures were crash, injury or mortality due to road traffic crashes. Two reviewers (MT and ZT) independently performed a risk of bias assessment of screened studies using the Effective Public Health Practice Project (EPHPP) tool (17). Any disagreement in this step was resolved by discussion to reach a consensus or deferred to a third author (DC) for the remaining cases (see Supplementary Appendix A3). In this step, we included controlled trial, case-control, cohort, quasi-experimental (interrupted times-series, before and after) study designs but excluded other study designs. In the next step, using a pre-defined data extraction form, two authors (MT and ZT) extracted variables of interest. We tested the data extraction tool with ten included model studies. Extracted variables are described in Supplementary Appendix A4. We classified interventions into seven categories using an adapted road safety intervention categories as defined in the WHO Save LIVES road safety technical package; see more details in Supplementary Appendix A5 (18).

The decision on the synthesis method was made after the risk of bias assessment to ensure that a sufficient number of studies were available reporting standardized metrics. Due to variability in statistical tests and different outcomes across studies, we decided to synthesize data using vote counting based on the direction of effect. We used a harvest plot to visualize the distribution of the evidence (19).

## Results

**FIGURE 1:**
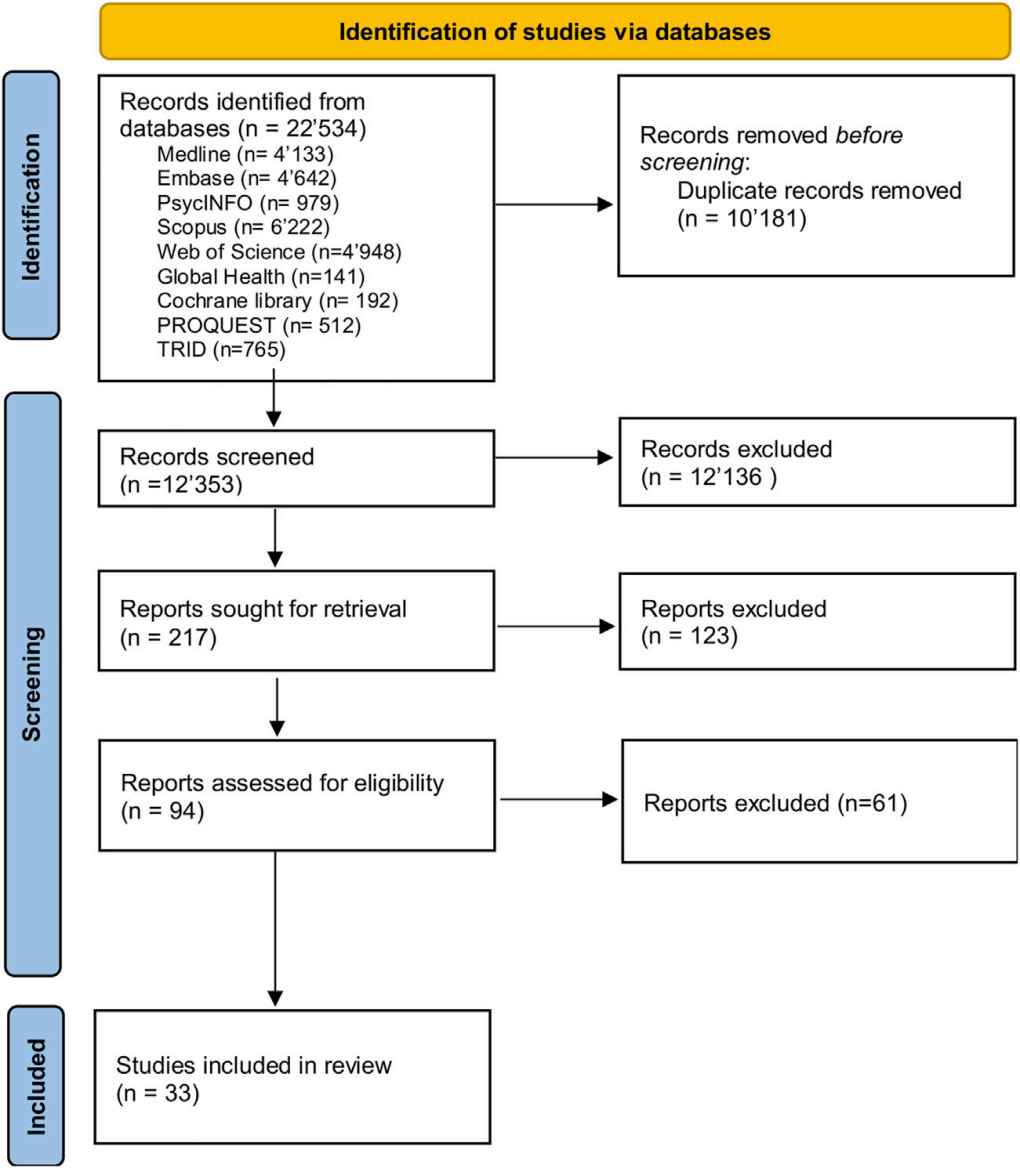
Study selection flow diagram. PRISMA, Preferred Reporting Items for Systematic Reviews and Meta-Analyses, for Road safety interventions in low and-middle income countries, 2011–2019.

**FIGURE 2:**
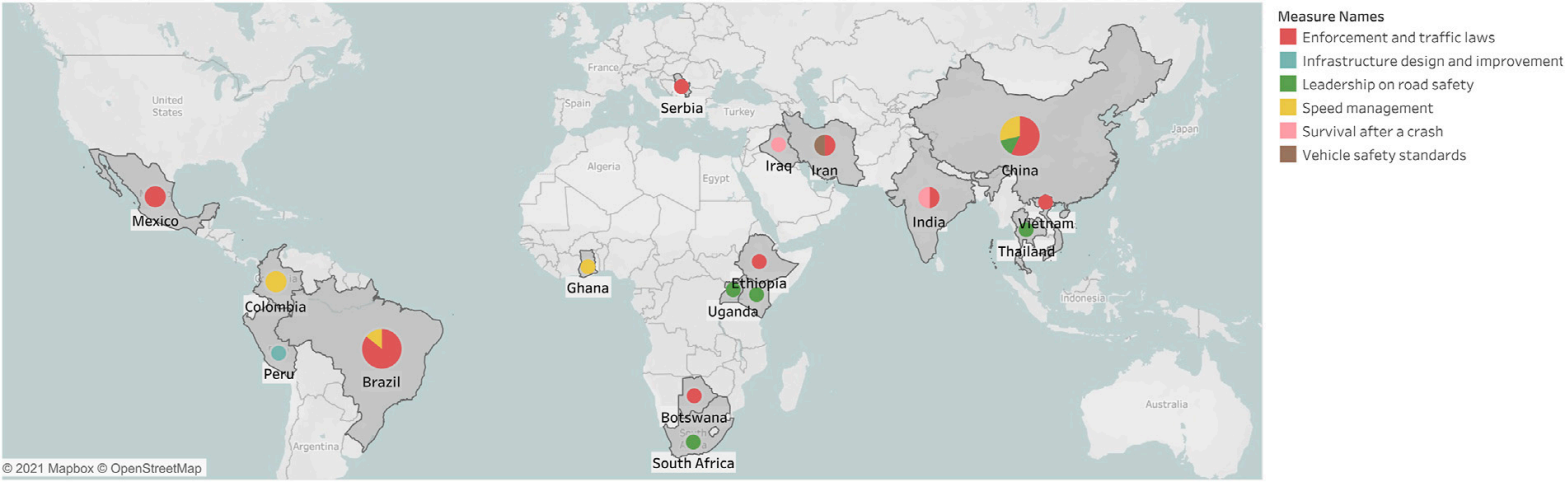
Geographical distribution of studies by type of road safety intervention in low and middle-income countries (systematic review, 2011–2019).

**FIGURE 3:**
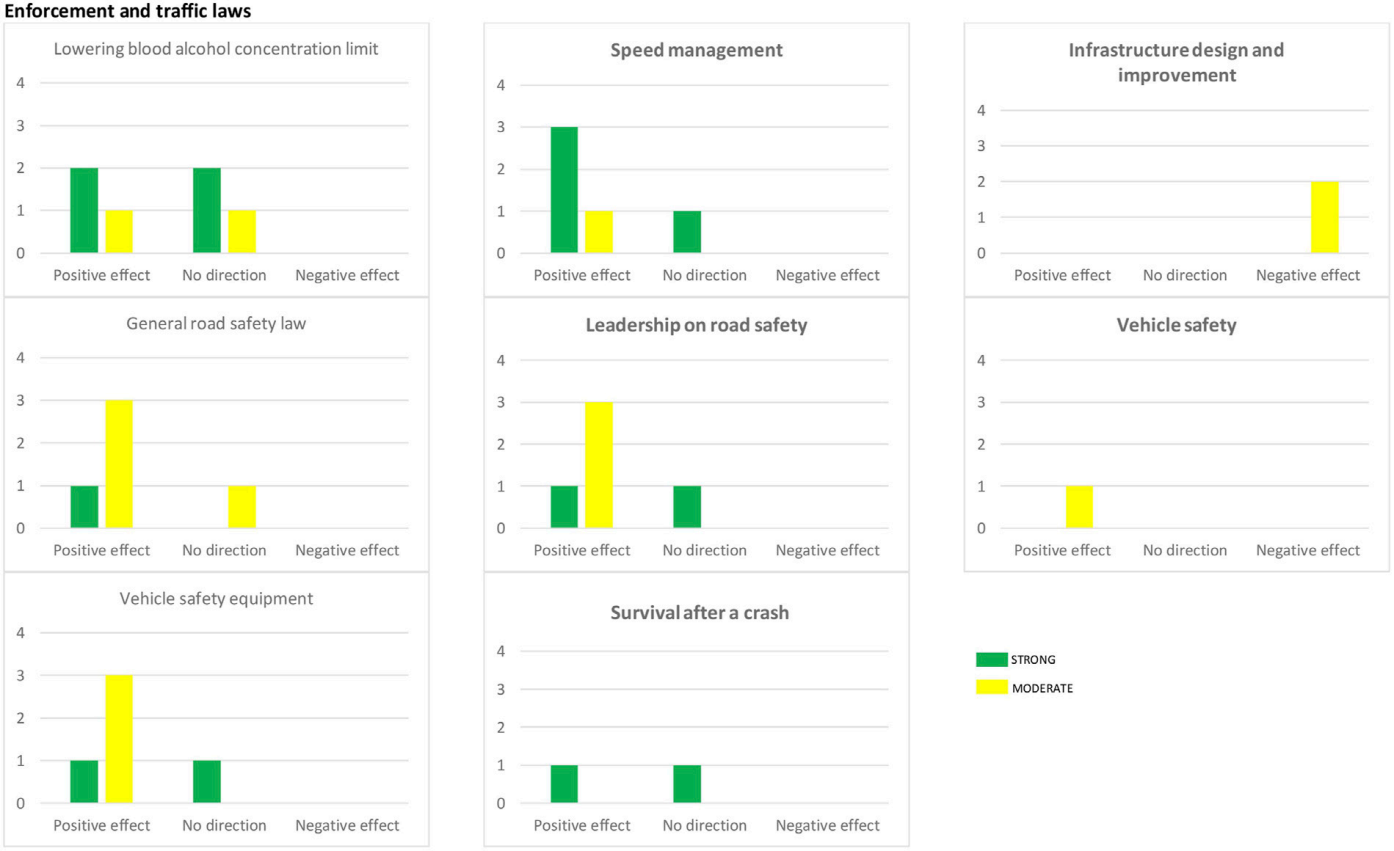
Summary of direction of health—related outcomes from included studies-harvest plot (systematic review, low and middle-income countries, 2011–2019). Distribution of the evidence for Six categories of interventions (Enforcement and traffic laws is presented with three subcategories) is plotted with the number of studies along the Y-axis. Each study is represented by a mark in each row for which that study had reported relevant results. Studies that rated STRONG using EPHPP quality appraisal tool are indicated with green bars, and studies rated MODERATE with yellow bars. The height of the bar indicates number of the studies.

The initial search retrieved 22,534 records. After deduplication, and screening for title-abstracts and full-text, using a predefined list of inclusion/exclusion criteria and assessment for risk of bias, 33 studies were included in the systematic review, of which 18 rated strong based on EPHPP quality appraisal tool as outlined in Figure 3 and Supplementary Appendix A3. Included studies were from 17 LMICs shown in Figures 1, 2. Twenty-seven studies were conducted in upper middle-income countries, four studies were conducted in lower middle-income countries, and two in low-income countries.

We organized the results around six categories by type of intervention as seen in Tables 1, 2. The categories with the most supporting evidence were enforcement and traffic laws, speed management, leadership (see harvest plot in Figure 3). Here we describe the range of effect in each category.

**TABLE 1:** Characteristics of included studies classified by the type of road safety intervention (systematic review, low and middle-income countries, 2011–2019).

Subcategory	Country	Target population	Type of outcome	Intervention start	Study duration	Study design
**Enforcement and traffic laws**						
**Lowering BAC limit**						
Andreuccetti, G (2011) (20)	Brazil	All drivers	Mortality, Injury	2008	2001 Jan–2010 Jun	Interrupted time-series
Jomar, R (2019) (22)	Brazil	All drivers	Mortality	2008	1999–2016	Interrupted time-series
Volpe, Fernando Madalena (2016) (21)	Brazil	All drivers	Mortality	2008	1980–2013	Interrupted time-series
Guimarães, A (2019) (23)	Brazil	All drivers	Crash (Fatal)	2008	2000 Jan–2017 Dec	Time series
Xiong, Xiuqin (2019) (25)	China	All drivers	Mortality, YLL*	2011	2008–2014	Interrupted time-series
Zhao, Ang (2016) (24)	China	All drivers	Injury	2011	2009 Jan 1–2012 Dec 31	Interrupted time-series
Gómez-García, L (2014) (26)	Mexico	General population	Mortality, Injury, Crash		1999–2011	Time series
**Multi-faceted road safety law**						
Aney, Madhav S (2019) (28)	China	General population	Mortality, Injury, Crash	2003	2000–2007	Difference in difference
Sheng, R (2018) (27)	China	All drivers	Mortality	2004	2002 Jan–2010 Dec	Interrupted time-series
Chandran, A (2014) (29)	Mexico	General population	Mortality, Injury, Crash	2008	1999 Jan–2011 Dec	Interrupted time-series
Abreu, D. R. O. M (2018) (30)	Brazil	All drivers	Mortality	1998 and 2008	1980–2014	Interrupted time-series
Abegaz, T (2014) (31)	Ethiopia	General population	Mortality, Injury, Crash (PDO**)	2007	2002 Jan–2011 Dec	Interrupted time-series
Sebego, M (2014) (32)	Botswana	All drivers	Crash	2008	2004 Jan–2011 Dec	Interrupted time-series
**Child restraints law**						
Nazif-Muñoz, J. I (2018) (33)	Serbia	Children aged 0–3 and 4–12	Injury	2009	2004 Jan–2014 Dec	Interrupted time-series
Nazif-Muñoz, J. I (2018) (34)	Brazil	Children aged 0–8	Mortality, Injury	2010	2008 Jan–2014 Dec	Interrupted time-series
**Helmet law**						
Phung, D (2019) (35)	Vietnam	Motorcycle drivers and pillions	Mortality	2007	2005–2009	Interrupted time-series
Gupta, Amit (2013) (36)	India	Motorcycle drivers and pillions Population above 18 years old	Mortality, Injury	N/A***	2011 Jan–2012 July	Cross sectional (paired subgroup analyses)
**Seatbelt law**						
Soori, H (2011) (37)	Iran	Car occupants	Mortality, Injury	2005	2004–2006	Before and after
**Speed Management**						
**Establish new speed limit**						
Leitão, P. A (2019) (38)	Brazil	Motor vehicles	Mortality	2015	2010 Jan 1–2016 Dec 31	Interrupted time-series
**Traffic calming measures**						
Guo, Y (2015) (40)	China	Motor vehicles	Crash	2008	2008 Feb–2013 Dec	Cross-sectional
Liu, Pan (2011) (39)	China	Motor vehicles	Crash	2008	2007 Feb–2009 Dec	Observational before and after (comparison group)
Damsere-Derry, J (2019) (41)	Ghana	Motor vehicles	Mortality	N/A	2006–2011	Matched case-control
**Camera**						
Martínez-Ruíz (2019) (43)	Colombia	Motor vehicles	Crash	2012	2008–2014	Quasi experimental difference in difference
González, J. F (2016) (42)	Colombia	Motor vehicles	Crash	2012	2010–2013	Difference in difference
**Leadership on road safety**						
**Public awareness**						
Habyarimana, J (2011) (44)	Kenya	Matatu drivers and passengers	Mortality, Injury, Crash	2008	2006 Jan–2009 May	Randomized controlled trial
**Education**						
Ji, Y (2017) (45)	China	Seventh grade students (bicyclists)	Injury	N/A	Not Mention	Clustered randomized controlled trial
Woratanarat, P (2013) (46)	Thailand	Motorcyclists	Injury	2007	2007 Dec–2009 Jun	Retrospective cohort
Muni, K (2019) (48)	Uganda	Motorcycle taxi drivers	Crash	N/A	2017 Oct–2018 Jan	Cohort
Huang, J. Y (2019) (47)	South Africa	Uber taxi drivers	Mortality	2013	2010 Jan–2014 Dec	Quasi experimental difference in difference
**Post-crash response**						
**Trauma care**						
Murad, M. K (2012) (49)	Iraq	All types of road users	Mortality	2003	3 months	Non-randomized single-blinded design
Vasudevan, V (2016) (50)	India	All types of road users	Mortality	2005	2001–2010	Before and after
**Infrastructure design and improvement**						
**Traffic signals**						
Quistberg, D. A (2014) (51)	Peru	Vehicle drivers and pedestrian	Crash	N/A	2010 Oct–2011 Jan	Matched case-control
**Vehicle safety standards**						
**ABS antilock**						
Khorasani-Zavareh, D (2013) (52)	Iran	Car occupants	Crash	N/A	2007 Mar–2008 Mar	Historical cohort (telephone survey)

*YLL, years of life lost.

**PDO, property damage only.

***N/A, Not applicable.

**TABLE 2:** Summary of outcomes of road safety intervention (systematic review, low and middle-income countries, 2011–2019).

	Intervention	Author (year)	Indicator	Outcome	Point estimate (95% CI)
**Enforcement of traffic laws**					
**Lowering blood alcohol concentration (BAC) limit**					
Brazil	Legislation: Law n 11.705, known as the Lei Seca or Dry Law. Lowering BAC limit for drivers from 0.06 to 0.02 g/dl	Andreuccetti, G (2011) (20)	Change in average monthly mortality rate (state of Sao Paulo)	Mortality	β = −0.100, SE = 0.042, *p* = 0.020
			Change in average monthly mortality rate (capital of Sao Paulo)	Mortality	β = −0.104, SE = 0.032, *p* = 0.002
			Change in average monthly injury rate (state of Sao Paulo)	Injury	β = −0.705, SE = 0.304, *p* = 0.023
			Change in average monthly injury rate (capital of Sao Paulo)	Injury	β = −0.441, SE = 0.217, *p* = 0.044
	Enforcement: Strengthening punishment by distinction between administrative (fine and temporary driver’s license suspension) and criminal sanctions (full suspension of driver’s license and detention) based on BAC results	Jomar, R (2019) (22)	Percent change in pedestrian annual mortality	Mortality	percentage change = −0.5, *p* < 0.01
			Percent change in Cyclist yearly mortality	Mortality	% change = −0.1, *p* > 0.01
			Percent change in Motorcyclists yearly mortality	Mortality	% change = −0.2, *p* > 0.01
			Percent change in vehicle occupant yearly mortality	Mortality	% change = −0.1, *p* > 0.01
	There was no alternative response when a driver refused to take the test	Volpe, Fernando Madalena (2016) (21)	Change in mortality rate (Belo Horizonte)	Mortality	β = − 0.783, *p* = 0.238
			Change in mortality rate (Rio de Janeiro)	Mortality	β = 0.643, *p* = 0.746
			Changes in mortality rate (Sao Paulo)	Mortality	β = − 0.615, *p* = 0.416
		Guimarães, A (2019) (23)	change in fatal accidents	crash	β = −0.068, SE = 0.118, *p* = 0.565
	New Dry Law, Law n 12.760: Strengthening enforcement and more severe penalties for offenders	Guimarães, A (2019) (23)	change in fatal accidents	Crash	β = −0.184, SE = 0.029, *p* < 0.0001
	Brazilian National Traffic Council Resolution n° 432 Strengthening enforcement	Guimarães, A (2019) (23)	change in fatal accidents	crash	β = −0.187, SE = 0.027, *p* < 0.0001
China	Legislation: Introducing limits. Drunk driving BAC 0.02–0.08 g/dl and drunk driving BAC >0.08 g/dl	Xiong, Xiuqin (2019) (25)	Change in monthly YLL (years)	Mortality	β = −778.1 (−1355.1, −200.1), *p* < 0.05
			Change in monthly YLL (years) in urban areas	Mortality	β = −166.1 (−360.1, 28.1)
			Change in monthly YLL (years) in sub-urban areas	Mortality	β = −612.1 (−1156.1, −68.1), *p* < 0.05
			Change in monthly mortality	Mortality	β = −11.1 (−21.1, −1.1), *p* < 0.05
			Change in monthly mortality in urban areas	Mortality	β = −16.1 (−34.1, 6.1)
	Enforcement: severe penalties: BAC 0.02–0.08 g/dl results in suspension of the driver’s license for at least 6 months and a fine of 1000–2000 CNY (approximately $160–320) and BAC >0.08 g/dl results in five to 10 years suspension of driver’s license and prosecution for criminal offenses		Change in monthly mortality in sub-urban areas	Mortality	β = −10.1 (−20.1, 1.1)
		Zhao, Ang (2016) (24)	Mean percent change in daily injuries	Injury	% change = −9.6% (−12.8, −6.5)
			Mean percent change in monthly injuries	Injury	% change = −11.9% (−19.7, −4.0)
			Decrease in mean percent change in daily day-time injuries	Injury	% change = 6.5% (13.4, 5.8)
			Decrease in mean percent change in daily nighttime injuries	Injury	% change = 13.3% (19.3, 7.2)
Mexico	“Ley Salvavidas”	Gómez-García, L (2014) (26)	Change in monthly road traffic related mortality	Mortality	β = −0.031, SE = 0.749, *p* = 0.097
			Change in monthly alcohol-related road traffic mortality	Mortality	β = −5.654, SE = 2.393, *p* = 0.018
	Sobriety checkpoints with breathalyzer to identify alcohol-impaired drivers. BAC 0.05–0.08 g /dl, results in a fine of 150–200 days of minimum wage, (equivalent to 662.87 and up to $ 883.82). BAC 0.081–0.13 g/dl, in addition to the financial sanction, the vehicle is stopped. BAC> 0.13 g/dl, the driver is taken to court		Change in monthly road traffic related hospitalizations	Injury	β = −0.123, SE = 0.682, *p* = 0.3
			Change in monthly crash rate	Crash	β = −9.932, SE = 4.355, *p* = 0.023
**Multi-faceted interventions**					
China	Road Traffic Safety Law in 2003 and the amendment in 2011: Safety standards were set, third party liability automobile insurance was made compulsory, a penalty points system was introduced, driving after drinking was prohibited, and legal responsibility was automatically attributed to motorists involved in an accident with a pedestrian or non-motorized vehicle	Aney, Madhav S (2019) (28)	Change in accidents per 10,000 population	Crash	β = −3.814, SE = 0.882, *p* < 0.01
			Change in deaths per 10,000 population	Mortality	β = −0.167, SE = 0.045, *p* < 0.01
			Change in injuries per 10,000 population	Injury	β = −1.43, SE = 0.425, *p* < 0.01
			Change in ratio of death to accident	Mortality	β = 0.111, SE = 0.020, *p* < 0.01
			Change in ratio of injury to accident	Injury	β = 0.313, SE = 0.047, *p* < 0.01
		Sheng, R (2018) (27)	Change in fatality rate after enactment of the law in 2003	Mortality	−10.9% (−1.5, −19.5)
			Change in fatality rate after enactment of the law in 2010	Mortality	−7.4% (−2.8, −16.6)
			Change in fatality rate after enforcement of the law in 2004	Mortality	−18.9 (−10.5, −26.5)
			Change in fatality rate after enforcement of the law in 2011	Mortality	−19.2 (−11.3, −26.3)
Mexico	Iniciativa Mexicana de Seguridad Vial (IMSEVI) 1^st^ phase: Drink-driving enforcement and seatbelt and child-restraint campaigns (March 2008–December 2009	Chandran, A (2014) (29)	Change in monthly mortality rate (Guadalajara-Zapopan)	Mortality	Rate = −0.04, SE: 0.08, *p*: 0.576
			Change in monthly mortality rate (Leon)	Mortality	Rate = 0.00, SE: 0.12, *p*: 0.977
			Change in monthly injury rate (Guadalajara-Zapopan)	Injury	Rate = −2.21, SE: 1.98, *p*: 0.264
			Change in monthly injury rate (Leon)	Injury	Rate = 0.07, SE: 3.72, *p*: 0.985
			Change in monthly crash rate (Guadalajara-Zapopan)	Crash	Rate = −5.29, SE: 3.80, *p*: 0.164
			Change in monthly crash rate (Leon)	Crash	Rate = −12.21, SE: 3.11, *p*: 0.000
	Iniciativa Mexicana de Seguridad Vial (IMSEVI) 2^nd^ phase: Drink driving enforcement and legislation in first year, followed by the addition of seatbelt and child restraint campaigns (January 2010- December 2011)	Chandran, A (2014) (29)	Change in monthly mortality rate (Guadalajara-Zapopan)	Mortality	Rate = −0.12, SE: 0.08, *p*: 0.125
			Change in monthly mortality rate (Leon)	Mortality	Rate = −0.09, SE: 0.13, *p*: 0.474
			Change in monthly Injury rate (Guadalajara-Zapopan)	Injury	Rate = −1.92, SE: 1.56, *p*: 0.219
			Change in monthly injury rate (Leon)	Injury	Rate = −0.32, SE: 3.99, *p*: 0.937
			Change in monthly crash rate (Guadalajara-Zapopan)	Crash	Rate = −10.39, SE: 4.75, *p*: 0.029
			Change in monthly crash rate (Leon)	Crash	Rate = −5.73, SE: 2.63, *p*: 0.029
Brazil	Brazilian Traffic Code in 1998	Abreu, D. R. O. M (2018) (30)	Change in number of deaths (1980–1997)	Mortality	β = 0.62, *p* < 0.0001
			Change in number of deaths (1998–2007)	Mortality	β = 0.70, *p* = 0.059
	Defining the attributions of the different institutions linked to road traffic safety and establishing the general rules and fines		Change in number of deaths (2007–2014)	Mortality	β = −0.26, *p* = 0.742
			Change in number of deaths (1998–2014)	Mortality	β = −9.69, *p* < 0.0001
Ethiopia	Road safety law Including prohibition of cell phone conversation while driving, mandatory seat belt and motorcycle helmet use	Abegaz, T (2014) (31)	Change in monthly non-injury crashes per 10k vehicles	Crash	β = −5.096 (−8.14, −2.05)
					SE = 1.54, *p* < 0.01
	Amendment includes laws against excessive speeding, driving under influence and unsafe loading		Change in monthly mortality per 10′000 vehicles	Mortality	β = −1.96 (−3.31, −0.61)
					SE = 0.68, *p* < 0.01
	Enforcement: higher penalty rate including suspension of the drivers’ license and roadside random check up on regular bases		Change in monthly injuries per 10′000 vehicles	Injury	β = −1.49 (−3.47, −0.49)
					SE: 1.003, *p* < 0.05
Botswana	October 2008 30% levy on alcohol products April 2009 Road Traffic Act increasing penalties for road traffic offenses, including driving without a license, speeding, alcohol-impaired driving, and failure to obey traffic signs and signals	Sebego, M (2014) (32)	Change in overall crash before and after June 2009	Crash	RR = 0.89 (0.83–0.95)
			Change in overall crash before and after June 2010	Crash	RR = 0.88 (0.82–0.95)
	November 2012, increasing alcohol levy to 40%		Change in fatal crash before and after January 2010	Crash	RR = 0.81 (0.68–0.97), *p* = 0.001
			Change in single vehicle night-time fatal crash rate before and after March 2010	Crash	RR = 0.69 (0.51–0.93), *p* = 0.016
**Age appropriate child restraint law**					
Serbia	Serbian Law on Road Safety (SLRS) July 2009	Nazif-Muñoz, J. I (2018) (33)	Change in child occupant injuries (ages 0–3) per child population	Injury	IRR = 0.80, SE = 0.07
	Legislation				*p* < 0.05
	1) Mandatory use of child restraints for children up to 3 years old		Change in child occupant injuries (ages 4–12) per child population	Injury	IRR = 0.87, SE = 0.06
	2) Children age 4–12 should be buckled up in the rear seat when traveling in motor vehicles				P not significant
	3) Mandatory seat belt use for all passengers		Change in child pedestrian injuries (ages 0–12) per child population	Injury	IRR = 0.98, SE = 0.17
	Enforcement: Drivers who fail to comply with this legislation can be fined heavily or face imprisonment for up to 30 days				P not significant
Brazil	Mandatory child restraint legislation (CRL) in 2010. Drivers of motor vehicles responsible for installing age-appropriate child restraint systems (seats for infants under the age of 7 years and 6 months of age), and ensuring proper use of such devices. Enforcement: Drivers who fail to comply with this legislation can be fined $191.47 Brazilian Reals (∼60 US dollars) and have their vehicle impounded, which is nontrivial considering the monthly minimum wage in Brazil for 2016 was $ 880.00, or ∼228 US dollars	Nazif-Muñoz, J. I (2018) (34)	Change in monthly child injuries (ages 0–8) per 100′0000 motor-vehicles	Injury	Immediate effect: IRR = 0.82 (0.63–1.06)
					Gradual effect: IRR = 0.98 (0.97–0.99)
			Change in monthly child injuries (ages 0–8) per 100′0000 children 0–8	Injury	Immediate effect: IRR = 0.82 (0.63–1.07)
					Gradual effect: IRR = 0.98 (0.97–0.99)
			Change in monthly white child injuries (ages 0–8) per 100′0000 children 0–8	Injury	Immediate effect: IRR = 0.91 (0.66–1.24)
					Gradual effect: IRR = 0.97 (0.96–0.99)
			Change in monthly non-white child injuries (ages 0–8) per 100′0000 children 0–8	Injury	Immediate effect: IRR = 0.74 (0.53–1.03)
					Gradual effect: IRR = 0.99 (0.97–1.00)
			Change in monthly child fatalities (ages 0–8) per 100′0000 children 0–8	Mortality	Immediate effect: IRR = 0.61 (0.45–0.84)
					Gradual effect: IRR = 1.00 (0.99–1.01)
			Change in monthly white child fatalities (ages 0–8) per 100′0000 children 0–8	Mortality	Immediate effect: IRR = 0.48 (0.33–0.68)
					Gradual effect: IRR = 1.00 (0.99–1.02)
			Change in monthly non-white child fatalities (ages 0–8) per 100′0000 children 0–8	Mortality	Immediate effect: IRR = 0.87 (0.55–1.37)
					Gradual effect: IRR = 0.99 (0.97–1.01)
**Motorcycle helmet law**				
Vietnam	Legislation: In June 2007, the Government passed a stringent law, making the wearing of helmet compulsory for rider and passenger on all roads effective December 15, 2007	Phung, D (2019) (35)	Change in monthly PYLLs per 100000 population	Mortality	−18.1 (−23.4, −12.8)
	Enforcement: Offenders would face a fine of US$6–US$12, an equivalent to approximately 30% of an average monthly income				
India	Legislation: Motor Vehicles Act in 1988, which mandated universal helmet use with all MTVs	Gupta, Amit (2013) (36)	Change in risk of deaths	Mortality	OR = 0.65 (0.48–0.86)
			Change in risk of serious head injury (AIS for head>=3)	Injury	OR = 0.34 (0.26–0.45)
	Enforcement: Opposition from Sikh community, on religious grounds that forbade men to cover their hair with anything other than a turban, and Sikh women were supposed to keep their head covered, led to ineffective law enforcement. Women were completely exempted, because it was impossible for enforcement agencies to differentiate Sikh women from others of a different community		Change in risk of serious facial injury (AIS for face>=2)	Injury	OR = 0.87 (0.57–1.26)
			Change in risk of cervical spine injury	Injury	OR = 0.74 (0.54–1.06)
**Seatbelt law**					
Iran	Seatbelt enforcement for front seat passengers	Soori, H (2011) (37)	Change in fatal injuries 1 year before and 1 year after seatbelt enforcement	Mortality	−3.3%
			Change in fatal injuries 1 year before and 2 years after seatbelt enforcement	Mortality	−1.7%
			Change in non-fatal injuries 1 year before and 1 year after seatbelt enforcement	Injury	+3.8%
			Change in non-fatal injuries 1 year before and 2 years after seatbelt enforcement	Injury	+2.6%
**Speed management**					
**Traffic calming measures**					
Brazil	Establishing new speed limit	Leitão, P. A (2019) (38)	Change in annual percentage change in mortality 5 years before and 1 year after intervention	Mortality	−4.92% compared to −7.38%
China	Parallelogram-shaped pavement markings A type of Illusionary pavement markings. Drivers feel that the travel lanes are becoming narrow, and the car is moving faster than it really is	Guo, Y (2015) (40)	Change in vehicle-pedestrian crashes	Crash	B = −0.237, SE: 0.067, *p* > x2: 0.000
					CI: −0.368, −0.106
			Change in rear-end crashes	Crash	B = 0.053, SE: 0.027
					*p* > x2: 0.049
					CI: 0, 0.106
	Transverse rumble strips pavement markings	Liu, Pan (2011) (39)	Change in crash frequency	Crash	Index of effectiveness
	Utilized to warn drivers of potential hazard by causing a vibration or audible rumbling transmitted through the wheels into the vehicle				θ = 0.75, SD = 0.24
Ghana	Speed tables, Speed humps and Speed bumps	Damsere-Derry, J (2019) (41)	Risk of pedestrian fatalities in absence of speed calming measures	Mortality	OR: 1.78 (1.09–4.43)
Colombia	Traffic camera	Martínez-Ruíz (2019) (43)	Change in number of monthly crashes	Crashes	IRR: 0.996 (0.991–0.999), P: 0.045
	Fixed cameras for detecting traffic violations: driving over the speed limit, running through a red light signal, violation of stop signs or other traffic signs, violation of the traffic ban schedule, and blocking the pedestrian crosswalks		Change in monthly number of crashes with casualties	Crashes	IRR: 0.995 (0.989–1.001), P: 0.120
		González, J. F (2016) (42)	Change in total number of crashes	Crashes	Low traffic flow: B = 17.6, SE: 7.72, *p* < 0.001
					Med traffic flow: B = 9.35, SE: 3.47, *p* < 0.1
					High traffic flow: B = 2.85, SE: 3.20, *p* > 0.1
			Change in number of crashes with a result in material damages	Crash (PDO)	Low traffic flow: B = 18.06, SE: 6.85, *p* < 0.001
					Med traffic flow: B = 7.11, SE: 5.6, *p* > 0.1
					High traffic flow: B = 1.23, SE: 4.10, *p* > 0.1
			Change in number of crashes with a result in injuries	Crash (with injured)	Low traffic flow: B = 15.3, SE = 8.7; *p* < 0.1
					Med traffic flow: B = 6.5, SE: 4.10, *p* > 0.1
					High traffic flow: B = 4.09, SE: 3.59, *p* > 0.1
		**Leadership on road safety**					
**Public awareness**					
Kenya	Raising public awareness by motivating passengers to speak up against bad driving in mini buses	Habyarimana, J (2011) (44)	Change in number of annual accident claims rate for vehicles in treatment group compared to vehicles in untreated group	Crash	Intent-to-treat
					β = − 0.051, SE = 0.016, *p* = 0.01
			Change in number of annual accident claims rate for vehicles in treatment group compared to vehicles in untreated group	Crash	Instrumental variable estimates
					β = -0.075, SE = 0.023, *p* = 0.01
	Intervention included a total of five stickers, with both fear stimuli (graphic images of injuries) and simple text messages. The stickers (11*3 in. in size) were placed on the metal panel between a passenger window and the ceiling of the vehicle. Messages aimed at motivating passengers to speak up against bad driving with a lottery that rewards matatu drivers for keeping the stickers in place		Change in number of injury/deaths claims rate for vehicles in treatment group compared to vehicles in untreated group	Injury and Mortality	Intent-to-treat
					β = −0.04, SE = 0.012, *p* = 0.01
			Change in number of injury/deaths claims rate for vehicles in treatment group compared to vehicles in untreated group	Injury and Mortality	Instrumental variable estimates
					β = −0.060, SE = 0.017, *p* = 0.01
***Education***					
China	Two hours Lecture for seventh grade students about traffic safety knowledge, injury prevention and how to address injuries	Ji, Y (2017) (45)	Difference in incidence of bicycle injuries between control and intervention group	Injury	Intervention group = 9.14% control group = 14.54%
					*p* < 0.01
Thailand	Multi-facetted courses for motorcyclists; including	Woratanarat, P (2013) (46)	Percent change in injury rate after 15-h license course	Injury	β = −0.35, SE = 0.14
					OR = 0.70 (0.53, 0.92), *p* value = 0.012
	- 15-h license course for students and general riders		Percent change in injury rate after 30-h instruction course	Injury	β = −0.35, SE = 0.26
	- 30-h instruction course for trainers and dealer staff				OR = 0.71 (0.42, 1.18), *p* value = 0.184
Uganda	Safe Boda:	Muni, K (2019) (48)	Comparing risk of crash in trained and regular motorcycle riders	Crash	RR = 0.61 (0.39–0.97), *p* = 0.04
	The company provides multiphase road safety training, helmets, vehicle maintenance and basic first responder training to its drivers. It also provides hairnets to passengers who are concerned about contracting skin diseases from a shared helmet. Initially, the training for the drivers was provided by the Uganda police and the Uganda Red Cross Society. However, this has since been transitioned to a team of SafeBoda trainers who were trained by the Global Road Safety Partnership and the Uganda police and Red Cross. Newly recruited drivers are trained on traffic signs and symbols, traffic regulations, the SafeBoda code of conduct, emergency response, customer care and how to use the SafeBoda app SafeBoda drivers undergo regular refresher trainings				Risk difference = −0.04, CI = −0.08, −0.01
South Africa	Ride-sharing service with education for drivers	Huang, J. Y (2019) (47)	Change in weekly road traffic deaths in treated provinces vs untreated provinces	Mortality	β = − 0.008 (−0.010, 0.006), *p* < 0.001
**Post-crash response**					
Iraq	Training paramedics in health centers and Emergency Departments of the district hospitals along main roads	Murad, M. K (2012) (49)	Comparing number of deaths in treated and untreated areas	Mortality	Treated = 7.8% Untreated = 44.2%
	The Trauma Care Foundation (TCF) health provides training for paramedics in health centers and Emergency Departments of the district hospitals along main roads. Due to resource limitations, the training program in 2003–2005 targeted the most remote districts of the province				CI of difference = 24.8–48.3%
India	Emergency Medical Services (EMS)	Vasudevan, V (2016) (50)	Comparing number of deaths in treated and untreated areas	Mortality	β: 200.5, SE = 199.1, *p* = 0.317038
	108 Services is a privately run EMS in India that operates and offers services similar to EMS. It was started in the state of Andhra Pradesh on August 15, 2005, by a nonprofit organization. As of early 2015, this service was operational in 16 states and 2 union territories. 108 services guarantee to reach the victims within a defined time, dependent on the type of contract they have in place with that particular state. The EMS vehicles are required to have a trained paramedical staff on duty				
**Infrastructure design and improvement**					
Peru	Phased pedestrian signals	Quistberg, D. A (2014) (51)	Risk of crash in sites with stationary figure pedestrian signal compared to non-signalized sites	Crash	OR = 8.88 (1.32–59.6)
	One type alternated between a motion-less green or red figure and the other includes a countdown for both vehicle and pedestrian traffic with a moving pedestrian figure		Presence of transit police to regulate traffic	Crash	OR = 0.05 (0.004–0.60)
**Vehicle safety standards**					
Iran	Anti-lock braking system (ABS)	Khorasani-Zavareh, D (2013) (52)	Risk of crashes due to brake failure for ABS compared to conventional brake system	Crash	β = −0.55, SE = 0.19 (−0.92, −0.18)
					*p* value = 0.004

IRR, Incidence rate ratio; OR, Odds ratio; RR, Rate ratio; SE, Standard error; PDO, Property damage only crash.

### Enforcement and Traffic Laws

We identified 18 studies in this category including interventions related to lowering blood alcohol concentration (BAC) limits, multifaceted road safety laws and mandatory use of safety equipment law.

#### Lowering BAC Limits

Of seven studies evaluating the effects of lowering BAC limits, four studies showed positive effects (see Figure 3). Brazil reduced the BAC limit for drivers and strengthened punishment by introducing the “Dry Law” (Lei Seca) in 2008. A 16% decrease in the number of mortality in Sao Paulo was reported 2 years after enactment of the Dry Law (20). However, looking at the trends of mortality rate, Volpe et al. found no significant change 5 years after enactment of the law (21). Despite the absence of any abrupt change in health-related outcomes, the law’s enforcement strengthened the pre-existing downward trends of mortality in some road users such as bicyclists and motorcyclists and pedestrians (22). Enabling effective enforcement of the law in 2012 led to a minor, yet steady decline in fatal crashes (23). Likewise, in China, enforcement of the criminal amendment for drinking and driving to the road safety law strengthened the pre-existing reduction trend in mortality and injuries (24, 25). Although enforcement of the law was better in cities, the reduction in years of life lost (YLL) was more evident in suburban areas (25). After enforcing the Life-Saving Law (“Ley Salvavidas”) in Mexico, a reduction was observed in the number of crashes as well as a downward trend in alcohol-related injury and mortality, but the authors could not find a strong association between the enforcement of the law and health-related outcomes (26).

#### Multifaceted Road Safety Laws

We found six studies evaluating the effects of multifaceted road safety laws, of which three showed positive effects (see Figure 3). In China, the road traffic safety law was enacted in 2003. Sheng et al. and Aney et al. found that while there was a reduction in the number of crashes and casualties, the law was more effective in preventing less severe crashes (27, 28). Similar results were observed in Mexico where a two-phase road safety initiative called IMESEVI—Iniciativa Mexicana de Seguridad Vial—was launched. Although there was no significant difference in injuries and mortality compared to the period preceding the implementation of the law, a reduction in the rate of crashes after the second phase of the intervention was reported (29). Enactment of the Brazilian Traffic Code in 1998 contributed to a reduction of 9.69 death per year from 1980 to 2014 with the highest decline among young adults (30). Similarly a multi-faceted road safety law in Ethiopia was followed by a rapid decrease of 19.2 and 12.4% in property-damage-only crashes and casualties respectively (31). And finally, in Botswana, a 30% levy on alcohol products complemented by the road traffic act contributed to a decrease in road traffic crashes and deaths, although the results were not sustained over time (32).

Taken together the results suggest that introducing a multifaceted road safety law was more effective on less severe crashes. However it is important to bear in mind this category includes various types of road traffic laws which impact health-related outcomes through different mechanisms, therefore results should be interpreted with caution.

#### Mandatory Use of Safety Equipment Laws

Four out of six studies in this category showed positive effects following the intervention (see Figure 3). In Serbia and Brazil, mandatory use of age-appropriate child restraints resulted in a reduction of injuries up to 8% among children (33, 34), but in Brazil the law had no effect on non-white children (34).

Two studies investigated the effects of a motorcycle helmet law on health outcomes—both emphasizing the diversity of outcomes among grographical and population subgroups. In Vietnam, law imposing severe penalties for offenders, resulted in a downward trend in potential years of life lost (PYLL) in 42 of the 61 provinces. Likewise, at national level a gradual reduction in PYLL (−18.1 per 100,000 person-months) was observed 6 months after the enactment of the law (35). In India, Gupta et al. studied the influence of the cultural setting that exempted women and Sikh men from wearing a helmet on road safety. Assessment of 224 male driver-female pillion crashes, revealed that women had lower compliance with the law (4.17% F, 61.8% M), and men had a 56 and 58% lower risk of deaths and serious head injury, compared to female counterparts (36).

Finally, in the case of seatbelt legislation we found one study from Iran. The results indicated with the increase of seatbelt use, a decline was observed in the severity of injuries in the first and second year after introducing the law (37).

### Speed Management

We identified four studies evaluating the effects of speed management interventions, generally in favour of the interventions (see Figure 3). Establishing a new speed limit in Brazil strengthened the downward trend in the annual change in mortality after the intervention (38). Traffic-calming measures, another speed management intervention, mainly targeted safety of pedestrians. Two studies showed that transverse rumble strips and parallelogram-shaped pavements before pedestrian crosswalks are likely to reduce vehicle-pedestrian crash frequency by 25 and 21% respectively (39, 40). Likewise, the presence of speed tables, speed humps and speed bumps, was associated with a lower risk of pedestrian fatality (41). Meanwhile, fixed cameras detecting traffic violations in Colombia resulted in mixed effects: from a 57% increase in the total number of crashes and crashes with injury in the intervention group to a 5–6% reduction in the number of crashes (42, 43).

### Leadership on Road Safety

We identified five studies evaluating the effects of education and public awareness interventions on road safety, four of which reported positive effects (see Figure 3). An initiative focusing on raising awareness among passengers and drivers of long-distance minibuses—Matatu in Kenya resulted in a 7.5 and 6% reduction in property-damage-only crashes and crashes with casualties respectively (44). Education initiatives were found to have minor to moderate effects on health-related outcomes. In China, an education initiative about bicycle safety for seventh-grade students reduced the incidence of bicycle injuries in children (45). Similarly, in Thailand, a safety-riding program targeting motorcyclists, reduced motorcycle-related injuries by 30% (46). Two studies from Uganda and South Africa showed that safety training and the provision of safe equipment through ride-sharing programs results in a reduction in crash risk (47, 48).

### Survival After a Crash

We found two studies showing different results in this category (see Figure 3). In Iraq, the presence of prehospital trauma care was associated with an 8% deaths rate compared to 44% in absence of pre-hospital trauma care (49). In contrast, no significant change in the number of road traffic deaths was observed after the introduction of the central emergency medical service in India (50).

### Infrastructure Design and Improvement

The most striking result to emerge from this category is the increase in the risk of pedestrian-vehicle collisions in presence of motionless green/red light signalization for pedestrian and vehicles. Whereas a negative association was found between police presence and the number of crashes in signalized intersections compared to non-signalized intersections (51).

### Vehicle Safety Standards

We found only one study evaluating the effect of the antilock braking system (ABS). The results showed that vehicles with antilock braking system are less likely than vehicles with a conventional braking system to be involved in road traffic crashes due to brake failure (52).

## Discussion

Our study aimed to systematically review the recent evidence on the effectiveness of road safety interventions in LMICs in light of principles of the Global Plan for the First Decade of Action for Road Safety. Eighteen out of 33 studies evaluated interventions in legislation and enforcement category, while some components of the road traffic system such as vehicle safety standards and road infrastructure and design were understudied. In most studies, only single interventions were assessed, omitting discussions around the complexity of road traffic system, relevant contextual factors, and its influence on the performance of interventions.

Our findings were in line with those of previous reviews, showing that most studies focused on changing road users’ behavior via enforcement, traffic laws, education and public awareness (9, 11, 53). Therefore, despite the recommendations of the Decade of Action to shift the burden of responsibility from road users to designers of the system, the role of designers in the system such as policy-makers, road managers, police, politicians, health sector, education system and etc. remained ignored (3). Our review exposes the scarcity of robust scientific evidence for some aspects of road safety—such as road infrastructure and design and vehicle safety standard—gaps that were revealed in previous literature and have remained unchanged after the Decade of Action (10, 11).

Another major finding of our review was that the existing scientific evidence in LMICs was focused on single interventions while little is known about the interaction between those interventions and components of the road transport system. This implies that the evidence around road safety is driven by component-oriented approaches while contextual factors are overlooked and road users are assumed to be the sole cause and changeable component in accidents (54). However a system is not merely a collection of single components (55) and road transport systems have characteristics of complex adaptive systems (56). In these systems, the relationships among the different components or stakeholders, emergent behaviours in the system, and contextual and historical factors are essential to improving performance (55, 57). In our review, single interventions were found to have short-term impacts, and their positive effect decreased over time. Salmon and Lenne argued that only focusing on one component of the system in isolation and ignoring other relevant factors will result in a diminished impact of the intervention (54). Furthermore, single interventions are less effective unless complemented with other interventions (58); Bambach et al, found that cumulative benefit of a combination of road safety interventions such as roadside barriers, helmet use, speed management and ban of alcohol consumption, resulted in synergies and a stronger positive effects (59).

Regarding evidence production, only 17 out of 138 LMICs were represented in our review. In line with previous literature, we found that a country’s income level was directly associated with representation in the production of scientific evidence: the number of studies from low-income and lower-middle-income countries was limited to 2 and 4 out of 33 studies respectively. These results reflect those of Perel et al. who also found that only 6 out of 236 road safety studies included in Cochrane systematic reviews were conducted in LMICs (60). Furthermore Zou et al. showed that among the top ten countries accounting for the production of 80.56% of road safety literature, only one LMIC—China—exists (61). This underrepresentation may partly be explained by factors related to the process of conducting research in LMICs and scientific publication systems (62). To measure the effectiveness of an intervention, researchers need information about characteristics of crashes, but many LMICs do not have a reliable information system recording this information (6). Another important factor affecting the conduct of research is the limited global funding opportunities for road safety. The available funding are donor driven and are dominated by HICs (63). The under-representation of LMICs in research publications goes beyond road safety research. In terms of the publication process, researchers in LMICs also face challenges. A review of the editorial boards of 27 global health journals showed that only 24% of editors were from LMICs and there was no editor-in-chief based in a low-income country (64). This lack of representation could be the result of institutional racism in publication process (65). In addition, authors from LMICs often cannot afford publication fees and consequently publish in journals that are not indexed in European or American (“global”) databases. This disparity leads to so-called “Academic-colonialism,” where the scientific publication system tends to empower researchers from HICs (66).

Finally, as mentioned before, the Global Plan for the Decade of Action encourages countries to set priorities for vulnerable road users—pedestrians, cyclists and motorized two and three-wheelers—accounting for half of the road traffic deaths globally (67). These users mostly benefit from speed management, alcohol control and enhanced visibility measures (68). In our review, we found 16 studies evaluating interventions such as lowering BAC limit, traffic calming measures, traffic signals and mandatory helmet law directly target vulnerable road users. However, we did not find studies evaluating the impact of proven effective interventions such as alcohol ignition interlock (69), street lighting (70), conspicuity aids and separate bicycle lanes (71) for vulnerable road users.

Selecting LMICs as the population for this review was both a strength and limitation of our study. We acknowledge that LMICs are very diverse in terms of resources and other contextual factors that might affect the subject of this study, however we used this term for general characteristics of countries in these income levels and to highlight the gaps in the literature among these contexts.

### Conclusion

Our study has shown the lack of a systems lens in evaluating road safety interventions in LMICs. We found that the same components of the system are often studied in isolation and little is known about the interactions between different components at a systemic level. The majority of the interventions are trying to change road users’ behaviour with education, legislation and enforcement and there is very little focus on eliminating hazards from the road system. A holistic understanding of the road transport system requires shifting from the prevailing paradigm of “fix the road user” to the systems thinking approach to “fix the system” while accounting for synergies and interactions among system components. A prerequisite in the application of systems approach in road transport systems research and practice is the availability of a good quality crash data system. This entails collaborating with relevant stakeholders and taking steps to improve road safety data systems. Until countries have a strong information system to collect various crash characteristics, the integration of the different information systems and using methodological approaches accounting for missing data will be essential. We are currently investigating ways of improving road traffic death registration systems in LMICs using systems thinking approaches such as process mapping and modelling, social network analysis (72).

Addressing the evidence gap in scientific publications from lower-income countries is a long-term but necessary process. It requires capacity strengthening by creating research networks that reach also into LMICs. The scientific community needs to give more visibility to evidence from LICs led by local scientists, by providing funding opportunities and facilitating publication processes to collect good quality evidence.

With the beginning of the second Decade of Action for Road Safety in 2021, we expect our findings to be valuable for informing global and national efforts towards designing inclusive, safe, resilient and sustainable societies, as well as the comprehensive research agenda needed to support such practice. In light of our findings, we recommend taking a step further from component-oriented approaches to applying systemic approached including systems thinking approaches in research and practice. This entails efforts to study complexity of the system as a whole and identifying international, regional and local actors and processes to improve accountability for improving road safety in LMICs.
