# Supplementary material for: Evidence From the Decade of Action for Road Safety: A Systematic Review of the Effectiveness of Interventions in Low and Middle-Income Countries
Source: Public Health Rev. 2022 Feb 21;43:1604499. doi: 10.3389/phrs.2022.1604499 (PMC8900064; doi:10.3389/phrs.2022.1604499)
Supplement: Supplementary file 1 [file DataSheet2.PDF]

Appendix2. Search Strategy (Systematic review, low and middle-income countries, 2011-2019)

Electronic databases:

1. EMBASE via OvidSP,
2. MEDLINE via OvidSP,
3. PsycINFO via OvidSP,
4. The Global Index Medicus (WHO),
5. Scopus, Web of Science,
6. Cochrane Library,
7. PROQUEST,
8. Transport Research International Documentation (TRID).

Search dates: Search in all databases were conducted in August 2019

Restrictions on publication period: The results shown here have no restrictions for publication period. Before screening phase authors restricted the results to 2011 to 2019, known as "the decade of action for road safety". After retrieving the records from databases, the results were filtered accordingly in EndNote X9 and the number of retrieved records were adjusted by the year for PRISMA flow diagram.

Restrictions on language: Articles published in English, Spanish, Portuguese, French, and Persian.

Types of studies:

Exclusion criteria: Literature reviews, systematic reviews and meta-analyzes or comments

Inclusion criteria: We did not define specific inclusion criteria for type of study in our search strategy.

Search Strategy

Database: Ovid MEDLINE(R) ALL <1946 to August 07, 2019>

----

1 (Africa or Asia or Caribbean or West Indies or South America or Latin America or Central America or Eastern Mediterranean or Middle East).hw,kf,ti,ab,cp. (272539)

2 (Afghanistan or Albania or Algeria or American Samoa or Angola or Argentina or Armenia or Armenian or Azerbaijan or Bangladesh or Barbados or Benin or Belize or Bhutan or Bolivia or Bosnia or Herzegovina or Hercegovina or Botswana or Brasil or Brazil or Bulgaria or Burkina Faso or Burkina Fasso or Upper Volta or Burundi or Cabo verde or Cambodia or Khmer Republic or Kampuchea or Cameroon or Cameroons or Cameron or Camerons or Central African Republic or Chad or China or Colombia or Comoros or Comoro Islands or Comores or Mayotte or Congo or Zaire or Costa Rica or Cote d'Ivoire or Ivory Coast or Cuba or Slovakia or Slovak Republic or Djibouti or Dominica or Dominican Republic or Ecuador or Egypt or El Salvador or Equatorial Guinea or Eritrea or Eswatini or Ethiopia or Fiji or Gabon or Gabonese Republic or Gambia or Georgia or Georgian Republic or Ghana or Grenada or Guatemala or Guinea or Guinea Bissauor Guiana or Guyana or Haiti or Honduras or India or Indonesia or Iran or Iraq or Jamaica or Jordan or Kazakhstan or Kazakh or Kenya or Kiribati or Korea or Kosovo or Kyrgyzstan or Kirghizia or Kyrgyz Republic or Kirghiz or Kirgizstan or Lao PDR or Laos or Lebanon or Lesotho or Liberia or Libya or Madagascar or Malaysia or Malaya or Malay or Malawi or Maldives Mali or Marshall Islands or Mauritania or Mauritius or Mexico or Micronesia or Moldova or Moldovia or Moldovian or Mongolia or Montenegro or Morocco or Mozambique or Myanmar or Burma or Namibia or Nauru or Nepal or Nicaragua or Niger or Nigeria or North Macedonia or Pakistan or Papua New Guinea or Palestine or Paraguay or Peru or Philippines or Philipines or Philippines or Phillippines or Romania or Rumania or Roumania or Russia or Russian or Rwanda or Ruanda or Grenadines or Samoa or Samoan Islands or Sao Tome or Senegal or Serbia or Sierra Leone or Solomon Islands or Somalia or South Africa or South Sudan or Sri Lanka or Saint Lucia or St Lucia or Saint Vincent or St Vincent or Sudan or Suriname or Surinam or Syrian arab republic or Syria or Tajikistan or Tadzhikistan or Tadjikistan or Tadzhik or Tanzania or Thailand or Timor leste or Togo or Togolese Republic or Tonga or Tunisia or Turkey or Turkmenistan or Turkmen or Tuvalu or Uganda or Ukraine or Soviet Union or Union of Soviet Socialist Republics or Uzbekistan or Uzbek or Vanuatu or Venezuela or Vietnam or Viet Nam or West Bank or Gaza or Yemen or Yugoslavia or Zambia or Zimbabwe).hw,kf,ti,ab,cp. (2740088)

3 Developing Countries.sh,kf. (83789)

4 ((developing or less* developed or under developed or underdeveloped or middle income or low* income or underserved or under served or deprived or poor*) adj (countr* or nation? or population? or world)).ti,ab. (92299)

5 ((developing or less* developed or under developed or underdeveloped or middle income or low* income) adj (economy or economies)).ti,ab. (498)

6 (low* adj (gdp or gnp or gross domestic or gross national)).ti,ab. (233)

7 (low adj3 middle adj3 countr*).ti,ab. (13520)

8 (lmic or lmics or third world or lami countr*).ti,ab. (6570)

9 transitional countr*.ti,ab. (155)

10 or/1-9 (2903572)

11 exp accidents, traffic/ (41821)

12 (injur* or death*1).ti,ab. (1448557)

13 11 and 12 (19090)

14 road*1.ti,ab. (37557)

15 (traffic or transport* or driving).ti,ab. (602345)

16 (accident*1 or collision*1 or crash*2 or safety).ti,ab. (568740)

17 ((road*1 or (traffic or transport* or driving)) adj (accident*1 or collision*1 or crash*2 or safety)).ti,ab. (17778)

18 ((road*1 or traffic or transport* or driving) adj (injur* or death*1)).ti,ab. (2464)

19 13 or 17 or 18 (32570)

20 exp motor vehicles/ or ambulances/ or automobiles/ or motorcycles/ or off-road motor vehicles/ (19843)

21 (vehicle*1 or car or cars or automobile*1 or motor vehicle*1 or motorvehicle*1 or motor cycle* or motorcycle*1 or motor bike*1 or motorbike*1 or bicycle*1).ti,ab. (167849)

22 (road user*1 or cyclist*1 or pedestrian*1 or occupant*1 or passenger*1 or motor cyclist*1 or motorcyclist*1).ti,ab. (22060)

23 20 and (12 or 16) (7330)

24 ((vehicle*1 or car or cars or automobile*1 or motor vehicle*1 or motorvehicle*1 or motor cycle* or motorcycle*1 or motor bike*1 or motorbike*1 or bicycle*1 or (road user*1 or cyclist*1 or pedestrian*1 or

occupant*1 or passenger*1 or motor cyclist*1 or motorcyclist*1)) adj (injur* or death*1 or accident*1 or collision*1 or crash*2 or safety)).ti,ab. (18127)

25 23 or 24 (23090)

26 (road infrastructur* or road design* or traffic calming or pedestrian crossing or rumble* or chicane* or kerb extension* or speed bump* or speed breaker* or roundabout* or crash cushion*1 or road side obstacl* or roadside obstacl*or guard rail*1 or guardrail*1 or road pavement*1 or refuge island*1 or single carriageway*1 or road lighting or traffic light*1 or signal control* or high friction surfacing or skid resistant surfacing or anti skid surfacing or antiskid surfacing or (separate*1 adj2 lane*1)).ti,ab. (2601)

27 Air bags/ or child restraint systems/ or head protective devices/ or seat belts/ (7781)

28 (vehicle safety equipment*1 or car safety equipment*1 or vehicle safety standard*1 or seat belt*1 or seatbelt*1 or air bag*1 or airbag*1 or child restraint*1 or booster seat*1 or pre crash system*1 or precrash system*1 or crash avoidance or collision avoidance or antilock brak* or anti lock brak* or reverse automatic brak* or vehicle design*1 or crash protecti* or day time running light* or daytime running light or high mounted stop lamp*1 or (electronic stability adj (program*1 or control))).ti,ab. (5505)

29 27 or 28 (10328)

30 *automobile driving/ or aggressive driving/ or automobile driver examination/ or distracted driving/ or driving under the influence/ or road rage/ (15717)

31 (speed limit*1 or speedlimit*1 or speed camera*1 or speed control* or speed adaptation*1 or driving licens*1 or driver*1 licens*1 or Point record system*1 or penalty point*1 system*1 or ((novice or inexperience*1 or young) adj1 driver*1) or driver training*1 or dr#nk driving or (drink* adj2 driving) or alcohol interlock or alcohol impaire*1 driving or blood alcohol concentration or random breath testing or sobriety checkpoint*1 or helmet*1).ti,ab. (12880)

32 30 or 31 (25769)

33 ((Post crash or postcrash) adj1 response).ti,ab. (1)

34 (26 or 29 or 32 or 33) and (12 or 16) (16995)

35 10 and (19 or 25 or 34) (8545)

36 limit 35 to (comment or editorial or guideline or historical article or letter or meta analysis or news or newspaper article or "review" or "systematic review") (635)

37 35 not 36 (7910)

38 limit 37 to (english or french or persian or portuguese or spanish) (6703)
