# Supplementary material for: Evidence From the Decade of Action for Road Safety: A Systematic Review of the Effectiveness of Interventions in Low and Middle-Income Countries
Source: Public Health Rev. 2022 Feb 21;43:1604499. doi: 10.3389/phrs.2022.1604499 (PMC8900064; doi:10.3389/phrs.2022.1604499)
Supplement: Supplementary file 2 [file DataSheet4.PDF]

Appendix 4. List of extracted variables

We create a data extraction tool based on following identifiers and tested it prior to the final extraction. Two reviewers (MT-ZT) independently extracted data using the data extraction tool in Microsoft Excel spreadsheet about the following variables.

1. **Study's general information:** title, authors' names and contact details, journal, publication type and date, study ID, funding sources;
2. **Methods:** Study approach design, timeframe of study, setting (rural/urban);
3. **Population characteristics:** countries, type of road users, number (sample size);
4. **Road safety intervention:** type (based on five pillars of Global plan for the Decade of Action), purpose.
5. **Comparator:** such as no intervention or a different type of infrastructure;
6. **Outcomes**: type of health related outcomes (Mortality, injury or crash)
7. **Data collection tool/ data source**;
8. **Conclusion**: author's conclusions.
