# Supplementary material for: Evidence From the Decade of Action for Road Safety: A Systematic Review of the Effectiveness of Interventions in Low and Middle-Income Countries
Source: Public Health Rev. 2022 Feb 21;43:1604499. doi: 10.3389/phrs.2022.1604499 (PMC8900064; doi:10.3389/phrs.2022.1604499)
Supplement: Supplementary file 3 [file DataSheet3.PDF]

Study	Selection bias	Study design	Confounders	Data collection methods	Withdrawals and drop outs	Global Rating
Abegaz, T 2014	Yellow	Yellow	Green	Green	Green	Green
Abreu, D. R. O. M 2018	Green	Yellow	Yellow	Green	White	Green
Andreuccetti, G 2011	Red	Yellow	Yellow	Green	White	Yellow
Aney, Madhav S 2019	Yellow	Yellow	Yellow	Green	White	Green
Chandran, A 2014	Green	Yellow	Green	Green	White	Green
Damsere-Derry, J 2019	Green	Yellow	Green	Green	Red	Yellow
Gómez-García, L 2014	Green	Yellow	Green	Green	White	Green
González, J. F 2016	Green	Yellow	Green	Green	White	Green
Guimarães, A 2019	Red	Yellow	Yellow	Green	White	Yellow
Guo, Y 2015	Yellow	Red	Green	Green	White	Yellow
Gupta, A 2013	Yellow	Red	Green	Green	White	Yellow
Habyarimana, J 2011	Yellow	Green	Green	Yellow	Green	Green
Huang, J. Y 2019	Yellow	Yellow	Green	Red	White	Yellow
Ji, Y 2017	Green	Green	Green	Green	Green	Green
Jomar, R 2019	Red	Yellow	Green	Green	White	Yellow
Khorasani-Zavareh, D 2013	Green	Yellow	Green	Green	Green	Green
Leitão, P. A 2019	Green	Red	Green	Green	White	Yellow
Liu, Pan 2011	Yellow	Red	Green	Green	White	Yellow
Martínez-Ruíz 2019	Yellow	Yellow	Green	Green	White	Green
Muni, K 2019	Red	Yellow	Yellow	Green	Green	Yellow
Murad, M. K 2012	Yellow	Yellow	Red	Green	Green	Yellow
Nazif-Munoz, J. I 2018_B	Green	Yellow	Yellow	Green	White	Green
Nazif-Munoz, J. I 2018_S	Green	Yellow	Green	Green	White	Green
Phung, D 2019	Green	Yellow	Yellow	Green	Green	Green
Quistberg, D. A 2014	Green	Yellow	Green	Green	Yellow	Green
Sebego, M 2014	Red	Yellow	Yellow	Green	White	Yellow
Sheng, R 2018	Green	Yellow	Green	Green	White	Green
Soori, H 2011	Yellow	Red	Yellow	Green	White	Yellow
Vasudevan, V 2016	Yellow	Red	Yellow	Green	White	Yellow
Volpe, F. M 2016	Yellow	Yellow	Yellow	Green	White	Green
Woratanarat, P 2013	Yellow	Yellow	Yellow	Green	White	Green
Xiong, Xiuqin 2019	Red	Yellow	Green	Green	White	Yellow
Zhao, A 2016	Yellow	Yellow	Green	Green	White	Green

Appendix 3. Quality assessment using EPHPP. Green=Strong, Yellow= Medium, Red= Weak and White= Not applicable. The EPHPP assesses studies based on eight criteria: Selection bias, Study design, Confounders, Blinding, Data collection methods, Withdrawal and dropouts, Intervention integrity and analyses (this section is descriptive without score) and scores them as "strong, "moderate" or "weak" . Due to the nature of road safety interventions, the research team decided not to consider blinding criteria from the final scoring
