# Supplementary material for: Evidence From the Decade of Action for Road Safety: A Systematic Review of the Effectiveness of Interventions in Low and Middle-Income Countries
Source: Public Health Rev. 2022 Feb 21;43:1604499. doi: 10.3389/phrs.2022.1604499 (PMC8900064; doi:10.3389/phrs.2022.1604499)
Supplement: Supplementary file 5 [file DataSheet5.PDF]

Appendix 5.Classification of road safety interventions adapted from WHO Save LIVES technical package

Category	Interventions
**Speed management**	Interventions such as establishing and enforcing speed limit laws, traffic-calming measures and speed adaptation technologies in vehicles.
**Leadership on road safety**	Interventions related to strengthening the system such as improving data systems and creating lead agency as well as raising awareness and education programs.
**Infrastructure design and improvement**	Interventions with the aim to provide safe mobility for all road users, such vehicle-free zones, bicycle or motorcycle lanes, sidewalks and safe crossings for pedestrians.
**Vehicle safety standards**	Includes establishing and enforcing safety standards for motor vehicles such as seatbelt, airbag and Anti-lock braking system (ABS)
**Enforcement and traffic laws**	Includes establishing and enforcing road safety laws such as, drinking and driving, use of helmet, seatbelt and child restraints.
**Survival after a crash**	Interventions such as prehospital and trauma care systems and training first responders.
